# Novel knowledge-based system with relation detection and textual evidence for question answering research

**DOI:** 10.1371/journal.pone.0205097

**Published:** 2018-10-03

**Authors:** Hai-Tao Zheng, Zuo-You Fu, Jin-Yuan Chen, Arun Kumar Sangaiah, Yong Jiang, Cong-Zhi Zhao

**Affiliations:** 1 Graduate School at Shenzhen, Tsinghua University, Shenzhen, China; 2 School of Computing Science and Engineering, VIT University, Vellore, India; 3 Giiso Information Technology Co., Ltd., Shenzhen, China; National Institutes of Health, UNITED STATES

## Abstract

With the development of large-scale knowledge bases (KBs), knowledge-based question answering (KBQA) has become an important research topic in recent years. The key task in KBQA is relation detection, which is the process of finding a compatible answer type for a natural language question and generating its corresponding structured query over a KB. However, existing systems often rely on shallow probabilistic methods, which are less expressive than deep semantic representation methods. In addition, since KBs are still far from complete, it is necessary to develop a new strategy that leverages unstructured resources outside of KBs. In this work, we propose a novel Question Answering method with Relation Detection and Textual Evidence (QARDTE). First, to address the semantic gap problem in relation detection, we use bidirectional long-short term memory networks with different levels of abstraction to better capture sentence structures. Our model achieves improved results with robustness against a wide diversity of expressions and questions with multiple relations. Moreover, to help compensate for the incompleteness of KBs, we utilize external unstructured text to extract additional supporting evidence and combine this evidence with relation information during the answer re-ranking process. In experiments on two well-known benchmarks, our system achieves *F*_1_ values of 0.558 (+2.8%) and 0.663 (+5.7%), which are state-of-the-art results that show significant improvement over existing KBQA systems.

## Introduction

Question answering (QA) has long been an important research topic in natural language processing. A factoid QA system is designed to automatically answer a factoid question with concise and accurate answers about objective facts. In recent years, as large-scale knowledge bases (KBs) such as Freebase [1], YAGO [2], and DBpedia [3] have been developed, QA systems have started to use these KBs as important resources to access general knowledge in a clean and structured format. The development of a highly accurate knowledge-based question answering (KBQA) system could be beneficial in many fields, such as medical treatment, voice assistance and consumer self-service.

Generally, traditional KBQA systems rely on KBs for general knowledge, and the key procedure necessary for utilizing KB knowledge in a QA system is to resolve the equivalent KB representation of the given question, and collect answers by inspecting semantic paths over the KB. In such a procedure, topic entities of question are first located, and possible predicates are then extracted and used to infer an answer type and find compatible answers. The process of finding the corresponding KB predicates that best match the type of the answer entity is called *relation detection* in this paper.

There have been studies investigating relation detection to predict the expected answer type of a question [4–9]. However, some of these works [7–9] are based on traditional probabilistic models; they mostly focus on general relation detection situations and do not take the end task of KBQA into consideration. Meanwhile, some works [4–6] are proposed to utilize deep learning methods to solve the KB-specified relation detection problem, but few of their methods exploit the combinations of local information and long-distance information. Thus, how to detect the relation type from the question remains unsolved.

Moreover, due to the incomplete knowledge of KBs, an answer entity could have no reachable path by starting from the question entities. It is insufficient to answer questions only by finding compatible relation predicates. Along with structured knowledge contained in KBs, we utilize unstructured text to extract implicit facts as supporting evidence for the answer. Although there have been a few recent studies [4–6, 10] investigating the refinement of answers with the help of text resources, they mostly use lexical feature-driven methods and rarely infer answers with a confidence score directly from the text, in contrast to our approach. Therefore, we focus on exploiting a better textual evidence inference model in leveraging external text resources.

In this work, we propose a novel Question Answering method with Relation Detection and Textual Evidence (QARDTE). The relation detection model is built on a network with bidirectional long short-term memory (Bi-LSTM) and convolution building blocks. The model learns a multi-level question representation by the combination of local word information and long semantic dependencies. Meanwhile, inspired by the idea of machine translation, we apply a pre-trained network that learns from parallel phrases to improve the robustness against a wide diversity of expressions. Furthermore, to address the problem of KB incompleteness, we use external text resources to extract unstructured information to infer our candidate answers. We propose a neural network-based method with an attention mechanism to compose a semantic representation between the question and text. This method improves the semantic match between the question and external text statements, thus leading to better performance. It also improves the ability of the system to answer compositional questions, which is usually performed through explicitly solving complex logical dependencies in previous methods.

We evaluate our method on two famous benchmarks, WebQuestions and Free917. In these experiments, our joint model achieves average *F*_1_ values of 0.558 (+1.9%) and 0.663 (+3.6%), outperforming existing state-of-the-art models. We also achieve better results for questions with multiple relations compared than the baseline methods. The results further prove the effectiveness of our proposed relation detection model. Our main contributions are as follows:

1. We propose a Bi-LSTM-based relation detection model with multi-level question representation that better captures semantic type information from questions, especially in the case of multiple relations.
2. We propose a neural network model based on an attention mechanism to better compose semantic representations between a question and text; it also leverages unstructured text as interpretable evidence in inferring candidate answers.
3. We construct a re-ranking procedure with combined information from the identified question type and the extracted textual evidence. The combination leads to further performance improvement.

The remainder of this paper is organized as follows. First, we discuss related work. Then, we introduce the details of our proposed method. Next, we describe the experimental datasets and setup and analyze the results. Finally, we present a summary of this paper and discuss future directions for improvement.

## Related work

Typically, methods of performing a factoid QA task can be categorized into two types based on the data they use: (1) QA methods based on structured data and (2) QA methods based on unstructured data.

QA systems in the first category are commonly based on semantic parsing methods. Those QA systems learn a grammar and parse natural language into a semantic representation [7–9, 11]. However, generating such a parser is very expensive and requires many annotated training examples and grammar rules; moreover, the diversity of language representation (also called the semantic gap) between natural language and the structural knowledge stored in KBs is also a major unsolved problem.

To mitigate the semantic gap problem, efforts have been made to develop improved relation detection methods. Paralex [7] uses a weak supervised method to learn different phrase representations from an auto-labeled language corpus. However, Paralex learns only from the surface text representations of the KB predicates, and the results are difficult to adapt to more generalized text patterns. Sempre [8] defines specific λ-expressions to represent the grammar structure and logical meaning of a natural language sentence. The system first tries to parse a sentence into as many λ-expressions as possible and then simplifies those expressions with certain predefined logical induction rules. Sempre achieves logical reasoning but at the cost of a more complex and error-prone procedure when generating λ-expressions.

The QA systems in the second category are mainly based on information extraction methods. The QA systems retrieve a set of candidate answers from a KB using relation extraction [5, 9, 12–14]. Our work follows this line of research. Typically, information extraction relies on a logical language with predicates that are closely related to the KB schema, and a dictionary is constructed that maps relations to KB predicates. The problem is then reduced to one of generating candidate logical forms, ranking them, and selecting one as the final answer [9]. In such a logical representation approach, a QA system constructs KB queries for every input question, and KB queries are then executed on the KB to retrieve the final answer. Cui et al. [14] attempted to directly learn coarse-grained two-factor question templates from a large sentence corpus, but their method tends to match only inputs that are similar to the learned corpus, and thus has extremely low recall. AgendaIL [12] treats QA as an information retrieval problem. It retrieves answers from existing QA pairs or uses distant supervision to obtain information from a text corpus. Using textual evidence not only mitigates representational issues in relation extraction but also alleviates the data scarcity problem to some extent.

In recent years, neural network models have been extensively developed in this field [11, 15–19]. One example is SubGraph [11]. SubGraph treats the QA task as a problem of finding a reachable path between two question objects in a KB. During pathfinding, a neural network is used to predict the next-step actions from a KB entity node. In works based on memory networks [17–19], an attempt is made to encode sentence meanings in distribution representation and perform question reasoning by computing the similarities between a question and the stored memories. However, in these works, no explicit relation type or textual evidence information is provided in the answer inference step. Specifically, Xu et al. [5, 15] have applied a multicolumn convolutional neural network (MCCNN) model. MCCNN is an end-to-end neural network model introduced in relation detection to solve the QA problem. Xu et al. also incorporate external text for answer ranking. Compared with these existing methods, our proposed method not only learns from the local information of every word, but also takes advantage of integrating semantic information over a long distance. In addition, most of these methods develop only lexical features with text refinement, while the ability of inferring answers within text is weaker than our deep method. Furthermore, we explicitly compose features combining the information between the question relations and inferred answer in the re-ranking procedure. The combination may further improve the performance.

## Methodology

### System overview

Given a natural language question, we first decompose that question into a list of word segments. One or several of those segments are identified as *entity mentions*, which are linked to their corresponding entities in the KB through entity linking procedures. The *topic entity* is the main entity in the question. The basic work flow of a QA system is starting from the topic entity, following one or more possible relation links, and finding compatible answers under certain constraints.

**Fig 1:**
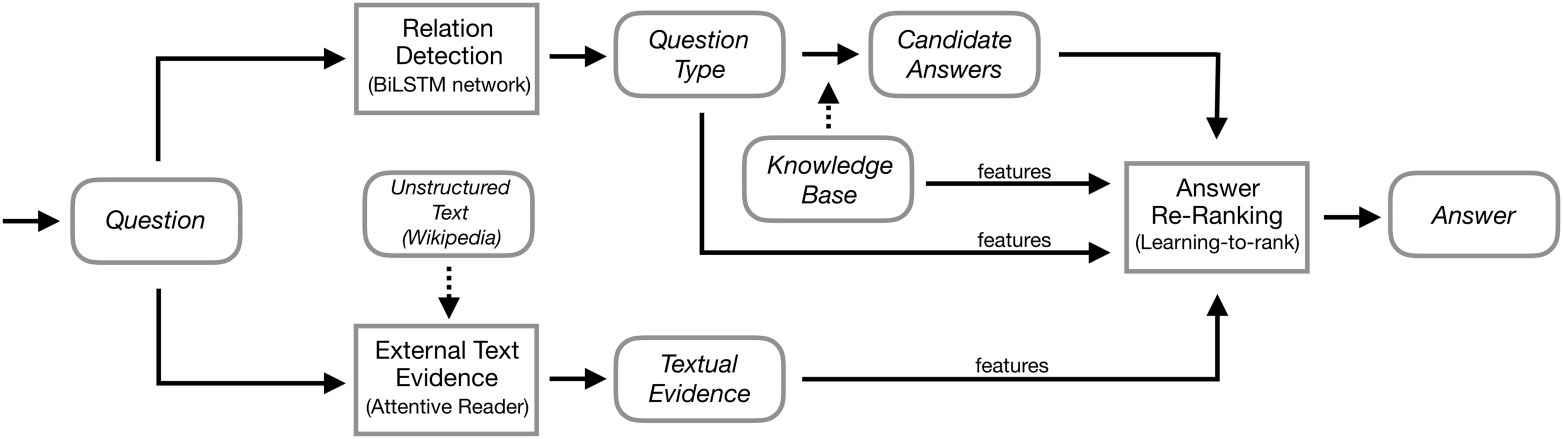
System overview. An input question text is first handled by a relation detector and an external text searcher; then, the features extracted from the question type information and the supporting textual evidence are combined to further rank the candidate answers.

Fig 1 gives an overview of our system implementation. We follow three main steps for processing an input question: (1) Relation Detection: perform relation detection on the question to obtain compatible relations and gather candidate answers over the KB; (2) Textual Inference: collect and infer supporting evidence from external unstructured text resources; and (3) Candidate Re-ranking: re-rank the candidate answers by combining the information extracted from both the KB and the external text.

Let us take a closer look at step 1. First, to generate possible candidate answers and the question type, we perform entity linking to identify the entities in the question and link tokens to possible KB entities with certain confidence scores. We then employ a relation detection method to predict the potential KB relations between the question and answer entities. With the identified entities and relations, we can make inferences over a KB and produce a list of candidate answers. Later, in step 2, we collect related text contents from an external text corpus. Specifically, in our experiments, we use Wikipedia as our text resource, and a search engine is built to retrieve related result snippets. We then use the proposed text inference model to further leverage text features from snippets that are semantically related to the question. Finally, in step 3, the candidate answers are re-ranked by combining the information obtained from the relation detection process and the extracted textual contents, thereby filtering out the answers that best fit the question.

### Entity linking

The goal of entity linking is to link the entity mention in a sentence to its corresponding entity in KB. The results of entity linking are further used by following relation detection and textual inference steps. We describe our entity linking method in this section.

First, we apply Stanford CoreNLP toolkit [20] to the question for word segmentation and part-of-speech (POS) tagging; those segments with noun (NN) or proper noun (NNP) tags are then treated as potential entities and further processed. Second, we construct an inverted index from string to entity by collecting all string expressions for every KB entity and splitting them into substring tokens. Meanwhile, we define the generation probability from a substring token to an entity by *p*(*entity*|*token*) as the corresponding confidence score for entity linking. The generation probability is estimated by the token frequency from the corpus; we adopt the pre-trained probability data from CrossWikis in work [13]. Finally, for a given word segment, every linked entity with a confidence score above a certain threshold is considered a possible match. Note that each word segment can have multiple matching KB entities. If adjacent word segments are linked to the same entity, then those segments are treated as a synonymous segment group.

Take the string “apple watch” as a simple example. The token “apple” can refer to Apple Inc. or just a red apple. Meanwhile, “apple” and “watch” can also be linked to the entity “Apple Watch”. Thus, we obtain 3 different entity links: (“apple”, Apple Inc.), (“apple”, red apple) and (“apple watch”, Apple Watch). These entity links are directly passed onto the next step without disambiguation; instead, they are jointly processed and ranked at the re-ranking step.

### Relation detection

The goal of relation detection is to predict the main relation between the topic entity and the answer from the question text. We describe our relation detection model in this section.

#### Problem formulation

Generally, KBs are represented in the form of (*subject*, *predicate*, *object*) triplets. The triplet is formally defined as *K* = (*e*_*sub*_, *p*, *e*_*obj*_), *e*_*sub*_, *e*_*obj*_, ∈ *E*, *p* ∈ *P*. *E* denotes the set of KB entities, and *P* denotes the set of KB predicates.

We formulate relation detection as a classification problem as in work [4, 5]. Given a decided KB predicate set *P* = {*p*_1_, *p*_2_, …, *p*_*K*_} of size *K*, a natural language question *q* in the form of word segments *q* = (*x*_1_, *x*_2_, …, *x*_*L*_) of length *L*, and its topic entity mention *m*, the objective of relation detection is to identify the KB relation connecting between the topic entity and the answer within the context of the question.

Specifically, the relation detection produces a probability distribution $\text{Pr}\left(p|q\right)\in R^{K\times 1}$ over relation set *P*. Every Pr(*p*_*k*_|*q*) denotes the possibility of predicate *p*_*k*_ being the correct relation for entity *m* in question *q*. We choose the predicate with the maximum probability ${\text{argmax}}_{p_{k}}\text{Pr}\left(p_{k}|q\right)$ as our predicted relation for the question.

#### Question encoder

**Fig 2:**
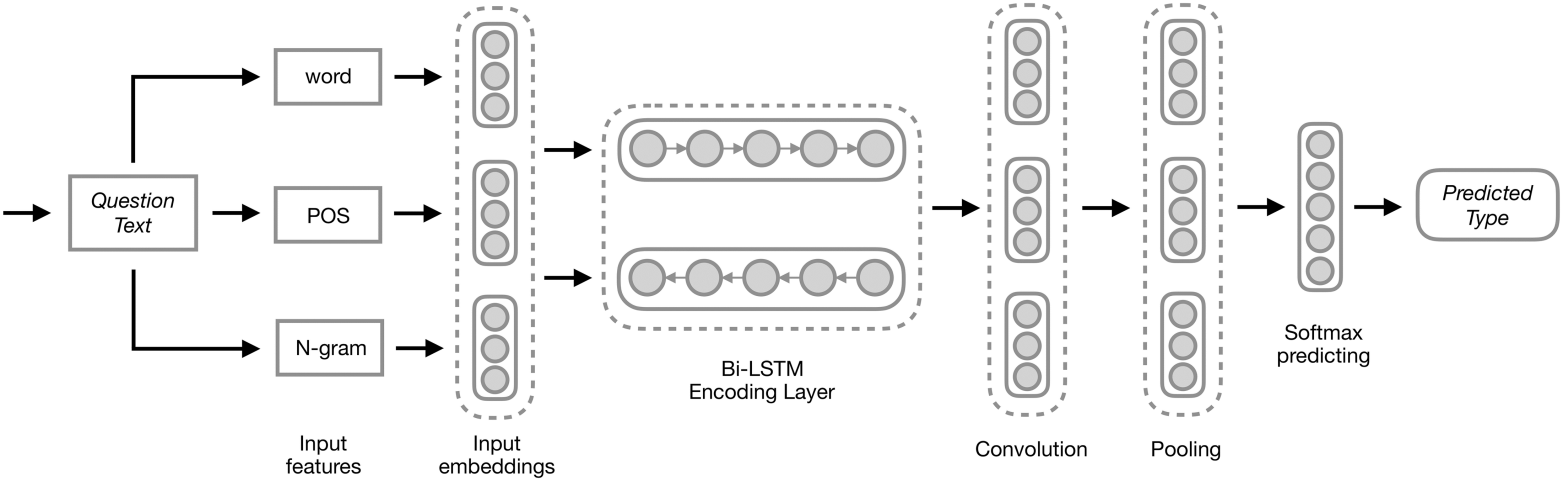
Graphical representation of our proposed Bi-LSTM model for relation detection. An input question is first encoded into vectors at different levels of abstraction, and the question type is then predicted from this vector representation.

Fig 2 illustrates the basic idea of our model. The input is the question *q*, and the output is the estimated probability distribution Pr(*y*|*q*) of relations for *q*. The question encoder transforms the input question *q* into a vector representation *v*.

First, we use a recurrent neural network (RNN) to capture the information of sequences of variable length. Specifically, to achieve a better question representation, a model with a Bi-LSTM [21] building block is built over the question *q*. Compared with a single LSTM layer, Bi-LSTM uses a second LSTM layer to receive a reversed data flow, providing the Bi-LSTM model with the ability to exploit context information in a time series.

Formally, let $u\left(x\right)\in R^{D\times 1}$ denote the feature vector of word *x*, and the input question is represented by a matrix *U* = {*u*(*x*_1_), *u*(*x*_2_), …, *u*(*x*_*l*_)}, $U\in R^{l\times D}$, where *D* is the size of a word vector. Each feature vector *u*(*x*) is composed by concatenating the vector space embedding of word *u*_*emb*_(*x*), $u_{emb}\left(x\right)\in R^{1\times D_{emb}}$, the POS tag index of word *u*_*pos*_(*x*), $u_{pos}\left(x\right)\in R^{1\times D_{pos}}$, the named entity tag of word *u*_*ner*_(*x*), $u_{ner}\left(x\right)\in R^{1\times 1}$, and the 2-gram information *u*_*bg*_(*x*), $u_{bg}\left(x\right)\in R^{1\times D_{bg}}$. These features reflect the meaning, context and fixed lexical properties of the word.

Let ${\vec{h}}_{l},{\overset{\leftarrow }{h}}_{l}\in R^{D_{h}\times 1}$ represent the forward and backward outputs of the Bi-LSTM network. As described by Graves [22], ${\vec{h}}_{l},{\overset{\leftarrow }{h}}_{l}$ are recursively computed as follows by

$$\begin{array}{c} \begin{array}{cc} {\vec{h}}_{l},{\vec{c}}_{l} & =\vec{LSTM}\left(u\left(x_{l}\right),{\vec{h}}_{l-1},{\vec{c}}_{l-1}\right)\\ {\overset{\leftarrow }{h}}_{l},{\overset{\leftarrow }{c}}_{l} & =\overset{\leftarrow }{LSTM}\left(u\left(x_{l}\right),{\overset{\leftarrow }{h}}_{l+1},{\overset{\leftarrow }{c}}_{l+1}\right) \end{array} \end{array}$$ (1)

where ${\vec{c}}_{l},{\overset{\leftarrow }{c}}_{l}\in R^{D_{h}\times 1}$ denote LSTM cell states. The final output of Bi-LSTM layer *h*_*i*_ is the concatenating of ${\vec{h}}_{i}$ and ${\overset{\leftarrow }{h}}_{i}$, $h_{i}=\left[{\vec{h}}_{i},{\overset{\leftarrow }{h}}_{i}\right]$. *h*_*i*_ represents the summarized information of the input sentence at the word *u*(*x*_*i*_).

By collecting every output of the Bi-LSTM time series, the input question is further represented by a hidden states matrix *H* = {*h*_1_, *h*_1_, …, *h*_*l*_}, $H\in R^{l\times D}$, where *D* is the size of word embeddings. Since Bi-LSTM has local consistency in the time series, we then utilize a convolution operation [23, 24] over the matrix *H* to exploit meaningful structure features. Let $m\in R^{k\times d}$ denote a 2D convolution operation. If applied to the position of question matrix *U*_*i*,*j*_, the 2D operator will cover a window of *k* words and *d* feature vectors; the window of convolution is denoted *H*_*i*:*i*+*k*−1,*j*:*j*+*d*−1_, and the output of the 2D convolution operator is denoted *o*_*i*, *j*_:

$$\begin{array}{c} o_{i,j}=f\left(m\cdot H_{i:i+k-1,j:j+d-1}+bias\right) \end{array}$$ (2)

where *m* is convolution function applied over the window of *H*. If the convolution operator moves 1 unit for each stride, then the range of *i* is from 1 to (*l* − *k* + 1), and the range of *j* is from 1 to (*D* − *d* + 1). After the convolution operator is applied to every possible window of the matrix *H*, a new feature matrix *O* is made:

$$\begin{array}{c} O=[o_{1,1},o_{1,2},\ldots ,o_{l-k+1,D-d+1}] \end{array}$$ (3)

where $O\in R^{\left(l-k+1)\times (D-d+1\right)}$. To generate encoded questions with different levels of abstraction, we apply a convolution layer containing filters with different sizes.

Meanwhile, to reduce the variance and compact low-level features, we apply an average pooling layer right after the convolution layers. Let *s*_*i*,*j*_ denote the average pooling operator; it will produce a new matrix *S* from *O*:

$$\begin{array}{c} S=[s_{1,1},s_{1,2},\ldots ,s_{l-k+1,D-d+1}] \end{array}$$ (4)

where *s*_*i*,*j*_ is the output of the average pooling operator $s_{i,j}=ap\left(O_{i:i+s_{1},j:j+s_{2}}\right)$, and the pooling operator is denoted $ap,ap\in R^{s_{1}\times s_{2}}$. Then, the *S* is a high level representation of the original sentence that summarizes all the information of words and structures in a sentence.

#### Relation prediction

For a question *q*, we use a softmax layer to compute the probability distribution Pr(*p*|*q*) from its vector representation *S* over its possible types:

$$\begin{array}{c} \text{Pr}\left(p|q\right)=Softmax(W_{sf}\left(S\right)+b_{sf}) \end{array}$$ (5)

where *W*_*sf*_ and *b*_*sf*_ denote the learned weight and bias of the softmax layer. We choose the predicate with the maximum probability ${\text{argmax}}_{p_{k}}\text{Pr}\left(p_{k}|q\right)$ as our predicted relation for the question.

#### Objective function and learning

To train the model, we use the categorical cross-entropy loss function between ground truths *t*(*q*) and network predictions *y*(*q*), and we define the objective function over all training data as follows:

$$\begin{array}{c} J\left(\theta \right)=-\sum_{q}\sum_{k=1}^{m}t_{k}\left(q\right)\text{log}\left(y_{k}\left(q\right)\right)+\frac{\lambda }{2}{∥\theta ∥}^{2} \end{array}$$ (6)

where *m* is the number of total target classes, $t\left(q\right)\in R^{m}$ denote the ground truth of the question *q*, *t*(*q*) is a one-hot vector. $y\in R^{m}$ is the output from the softmax layer and represents the estimated probability for every class. λ is the L2 regularization parameter, and *θ* is a penalty term of all model parameters. We train our model over shuffled mini-batches, and we use stochastic gradient descent with RMSProp [25] as the update strategy to minimize the objective function.

#### Pre-trained projection layer

Due to the semantic gap problem, even relations with similar semantic meanings have various textual representations. In addition to using CNN or RNN building blocks in the network design, we also propose a projection method that learns implicit transformations to further exploit the problem. It is inspired by Berant’s work [7], which uses paraphrases to learn a transition probability between different phrases, and the auto-encoders network [26] used in deep learning studies.

First, we adopt the Paralex monolingual paraphrase corpus from Berant [7] and then use the corpus to train an unsupervised auto-encoder [26]. The auto-encoder aims to learn synonymous expressions for every input within its implicit network values. After the convergence of the auto-encoder, we use it as an internal network layer embedded in relation detection networks. The layer projects an input phrase to its synonymous embedding vector.

#### Candidate answer generation

We generate candidate answers with a greedy generation strategy as follows. If an answer is chosen in any one step, then the answer will be marked as a candidate for later textual inference and re-ranking: (1) we first collect answers that basically match the relation and are connected to question entities; (2) we then collect answers that match the relation and can reach question entities within 2 hops; and (3) we select all answers that can reach question entities within 2 hops.

### Textual inference on Wikipedia

KBs contain a large amount of structured knowledge, but they are still far from complete. Due to the incompleteness of KB’s knowledge, an answer entity could have no reachable path by starting from question entities; therefore, it is insufficient to answer questions by finding only compatible relation predicates.

Our textual inference model is motivated by how people solve unknown questions by searching them through text corpus. Suppose that a person receives a question “When was Obama elected as president?”; to solve the question, the person first searches for text contents related to the sentence. After obtaining some text snippets, for example, a snippet “Barack Obama won the 2008 presidential election”, the person reads the snippet along with the question to infer the answer. Because the person can determine in advance what the question asked (“when”) and what the question is mainly discussing (“Obama”, “elected”, “president”, or the precise KB relation “government.politician.election_campaigns”), the person can pay more attention to certain words that match the question type and are highly related to the question content. Then, the person is able to infer that the answer to the question is “2008”.

Therefore, in this section, to help compensate for the incompleteness of KBs, we first obtain supporting textual evidence that may contain potential facts. Then, we introduce a textual inference model that learns to indicate the answer.

#### Collecting textual evidence

In this step, we need to locate text sentences related to the given question. Without loss of generality or simplicity, we use an English Wikipedia dump as our source of external knowledge instead of using a black-box commercial search engine. We remove all Wikipedia special language markup (https://en.wikipedia.org/wiki/Help:Wiki_markup) from the raw Wikipedia dump and then build a local search engine with the full text content from each extracted encyclopedia page.

During textual evidence collection, we first remove question words (such as when, what, how, …, etc.) and question marks from the original question and then provide the cleaned question as a search query to our local search engine; the search engine returns the retrieved Wikipedia pages ranked by the BM25 score function [27]. Then, we identify and collect key text snippets from every page by matching the topic entities with the question. Additionally, we use the Stanford CoreNLP toolkit [20] to perform text tokenization and POS tagging in all steps.

#### Attention model

**Fig 3:**
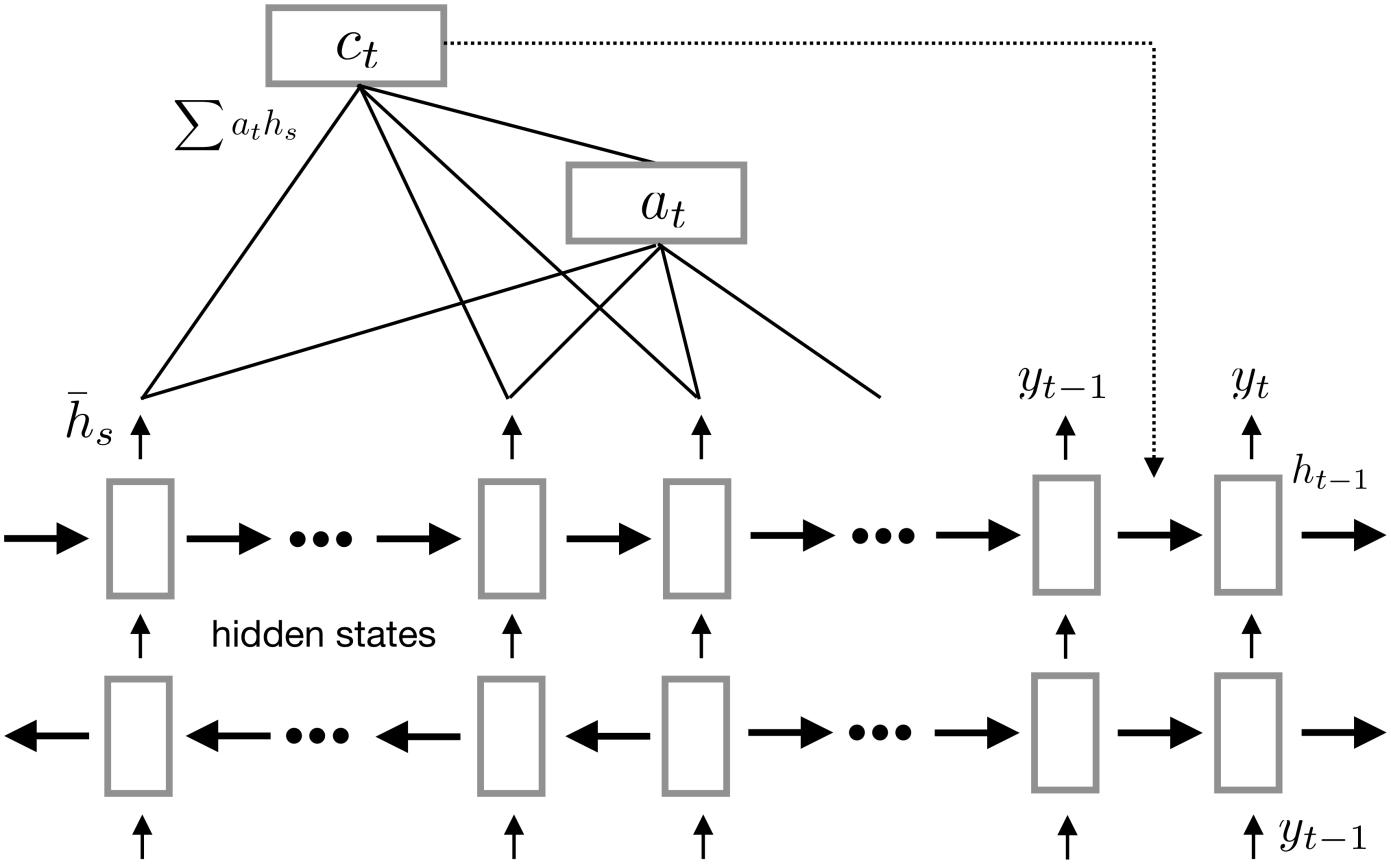
Attention mechanism used in our Bi-LSTM neural network.

If we know the question before we start reading a supporting snippet, we understand the text better if extra attention could be paid to those tokens semantically related to the question. Inspired by the document reader used in DeepMind [28], we introduce an attention mechanism to allow the contextual information from the retrieved snippet to influence the attention paid to each word. The attention mechanism is illustrated in Fig 3.

Our evidence extraction method is a classification problem, the input of the model is the question, the relation is extracted from the detection step, and the snippet is from the search results. The answer entities with the attention weight as their score are identified from the question word segments during the classification. If an answer entity is composed of multiple tokens, then we use the maximum attention weight among those word segments.

In our model, we first define the RNN’s (Bi-LSTM) hidden state *s* at time *i* as:

$$\begin{array}{c} s_{i}=f\left(s_{i-1},y_{i-1},c_{i}\right) \end{array}$$ (7)

where *s*_*i*−1_ is the previous hidden state, *y*_*i*−1_ is the previous output, and *c*_*i*_ is the context vector constructed by using the attention mechanism. The context vector is composed by summing and weighting the encoded words. It denotes the importance of every encoded word in predicting the word *i*. Formally, the context vector *c*_*i*_ is defined as:

$$\begin{array}{c} c_{i}=\sum_{j=1}^{T_{x}}a_{ij}h_{j} \end{array}$$ (8)

The attention weight *a*_*ij*_ denotes the weight of word *i* in predicting the word *j*. The attention weight is calculated from the encoded sequence *h*_*j*_ and the hidden state *s*_*i*−1_, and then normalized by the softmax operation:

$$\begin{array}{c} a_{ij}=\frac{\text{exp}(e_{ij})}{\sum_{k=1}^{T_{x}}\text{exp}\left(e_{ik}\right)} \end{array}$$ (9)

where *e*_*ij*_ denotes *e*_*ij*_ = *a*(*s*_*i*−1_, *h*_*j*_), and *a* is the attention function. In our model, we use a simplified attention so that the attention function *a* is defined as *e*_*ij*_ = *v*_*a*_ ⋅ tanh(*Wh*_*j*_ + *b*). The *e*_*ij*_ first passes the encoded sequence *h*_*j*_ through a vanilla neural layer, obtains hidden representation of the sequence, and then multiples it with a vector *v*_*a*_. *v*_*a*_, which is a vector representing the input sentence and is initialized during training.

**Fig 4:**
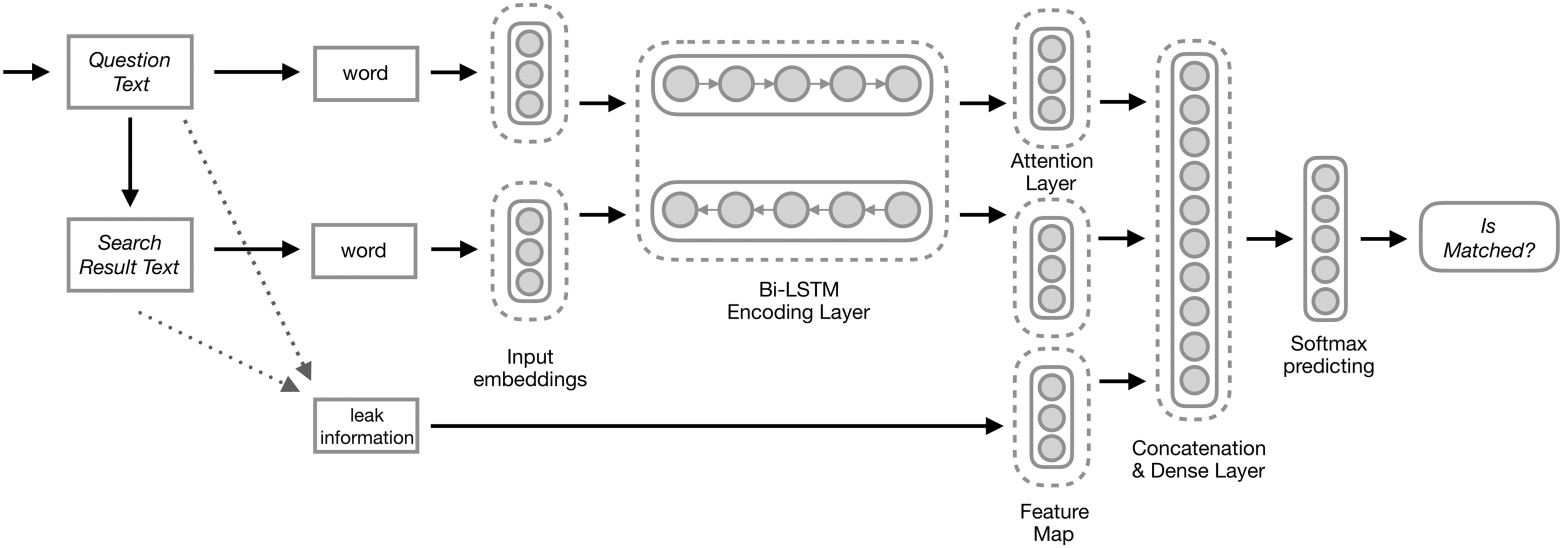
Graphical representation of our model with the attention mechanism for textual inference. The input of the model is the question, text snippet and detected question relation. The output is a Boolean (probability) value indicating whether the text snippet is the support evidence and contains the right answer.

Fig 4 illustrates the basic idea of our textual inference model. We apply this Bi-LSTM model with an attention mechanism over key snippets returned by the search engine. The model’s input is the question, detected relations from the question, and the text snippet. The detected relations are the prediction of our relation detection model. The leak information is composed of the detected relations and the position of the matched words between the question and snippets. The leak information reflects high-level supervision information between question and snippets. Meanwhile, all other input textual resources (question, text snippet) are transferred to word embeddings and are sent to a Bi-LSTM network layer. We setup the attention layer over the output of the Bi-LSTM layer. The output of the attention layer is concatenated with the leak information, and the concatenation is processed by a dense network layer. Then the output is sent to the softmax layer for normalization and prediction. The output of the model is the probability indicating whether the key snippet contains the answer and can be used as textual evidence to the corresponding question. In addition to the probability, we extract the attention score from the attention layer and further use them for textual features in the following section.

#### Composed textual features

We use the following statistical lexical features extracted from the Wikipedia pages and snippets. The input of this feature-generating procedure contains (1) a question; (2) a KB relation (from the relation detection procedure or the derived relations by collecting them from connected entities); and (3) a candidate answer (or phrase).

1. Overlap (Jaccard similarity $\frac{S_{A}\cap S_{B}}{S_{A}\cup S_{B}}$): (a) the ratio of how much of a page covers question entities; (b) the ratio of how much of a page covers answer entities; (c) the ratio of how much of a text snippet covers question entities; (d) the ratio of how much of a text snippet covers answer entities; and (e) the overlap between the question token set and the answer token set.
2. Boolean flags: (a) whether the question token set intersects with the token set from retrieved pages; (b) whether a retrieved page contains question entities; (c) whether a key text snippet contains question entities; (d) whether the number of entities from retrieved pages exceeds a threshold (20); and (e) whether the number of relations connected to the entity exceed a threshold (30).
3. Real number values: (a) the number indicating how many words matched between the question and its relation; (b) the number of possible entities linked to the string literal in the question; (c) the number indicating how many entities are present in retrieved pages; and (d) the TF-IDF value for entities.
4. TF-IDF vector between the question entities and answer entities: (a) cosine similarity between the question and a retrieved page; (b) cosine similarity between the question and the extracted relation; and (c) cosine similarity between the question and a key text snippet.
5. Features from the textual inference model: (a) attention score of every answer entity in the textual snippet; (b) whether the attention score of the entity exceeds a threshold (0.15); (c) cosine similarity between the attention words and candidate answers; (d) whether the relation type of the attention words match the question; and (e) whether the current text snippet is a valid supporting evidence.

We treat the normal textual features between the question and the retrieved passages as the global context, and the features from the key snippet as the local context. Similar to people skimming and scanning while reading, features from the global context are usually coarse-grained and represent a surface correlation between the question and articles, whereas, features from the local context are fine-grained and require many detailed evaluations between snippets and questions.

### Candidate re-ranking

This section describes the joint exploitation of the relation detection results and the extracted textual evidence. Inspired by learning-to-rank approaches for information retrieval [29, 30], for each question, we produce a final ranking of the candidate answers using the feature vectors described previously. The top-ranked candidate answer is then selected to provide the final answer.

To train the pairwise learning-to-rank classifier, for a question with *n* candidate answers, we randomly select half of the candidates. Then, for each randomly selected candidate *r*_*i*_ and the correct candidate *c*, where *r*_*i*_ ≠ *c*, we create two sample pairs, a positive one (*c*, *r*_*i*_) and a negative one (*r*_*i*_, *c*). The feature representation for a pair (*a*, *b*) is a tuple consisting of the individual feature vectors and their difference is computed as follows:

$$\begin{array}{c} \phi_{pair}\left(a,b\right)=\left(\phi \left(a\right)-\phi \left(b\right),\phi \left(a\right),\phi \left(b\right)\right) \end{array}$$ (10)

where *ϕ* is a function returning a vector containing all of the features in the previous section.

We use a forest of decision trees [31] as the classifier to be trained in the learning-to-rank procedure. Random forests are also simple, robust, and able to learn non-linear decision boundaries [13].

During the re-ranking procedure, for a candidate answer list *A* = [*a*_1_, *a*_2_, ⋯, *a*_*l*_], we compose a list for comparisons that contains all possible answer pairs as *A*′ = [(*a*_1_, *a*_2_), (*a*_1_, *a*_3_), ⋯, (*a*_2_, *a*_3_), ⋯, (*a*_*l*−1_, *a*_*l*_)]. We apply the trained classifier over those answer pairs. If the prediction of the classifier over pair (*a*_*i*_, *a*_*j*_) is 1, it indicates that the answer *a*_*i*_ is superior to answer *a*_*j*_, otherwise the answer *a*_*j*_ is superior to answer *a*_*i*_. Then, we obtain a sorted list of candidate answers.

#### Textual features

In addition to the common statistical textual features mentioned previously, we use N-grams as additional features. We collect 2-grams from the stemmed words in the question sentence (*w*_0_, *w*_1_, …, *w*_*n*_), in the form [(*w*_0_, *w*_1_), (*w*_1_, *w*_2_), …, (*w*_*n*−1_, *w*_*n*_)]; then, we count their frequencies to be used as features.

#### Relation features

Derived from the relation detection results discussed previously, two levels of relation matching measurements are used as our re-ranking features.

The first level is direct matching. A relation is another kind of entity in KBs, where each relation has its information type, which is the same as a normal entity. Therefore, we define a confidence score to measure how closely a relation string matches the original answer predicate. In detail, we set a score of 1.0 for complete matching, a value of 0.5 for fuzzy matching (includes the matching of derived KB relations), and a value of 0 for no matching.

The second level is semantic matching. Since it is insufficient to use only an arbitrary matching score and each relation is also composed of words, we leverage the textual words in each relation to calculate a matching score. We use the average embedded word vector that represents the relation itself:

$$\begin{array}{c} Rel\left(w_{0},w_{1},\ldots ,w_{n}\right)=\frac{1}{n}\sum_{i=1}^{n}Embedding\left(w_{i}\right) \end{array}$$ (11)

where we use the cosine similarity to measure the similarity between two relation vectors.

## Experiments

In this section, we introduce the experimental setup, the main results and a detailed analysis of our system.

### Experimental setup

#### Datasets

We adopt the WebQuestions dataset from Berant’s work [8]. The WebQuestions dataset is created by collecting real-world questions submitted to Google Search. The answers to the entries in WebQuestions are annotated manually by crowd-sourced workers. WebQuestions contains 5,810 question and answer pairs. As is typically done in similar works [8], we split WebQuestions into a training set (3,778 questions, 65%) and a test set (2,032 questions, 35%). In our experiments, we leave out 20% of the training questions as a development set. Free917 [32] is another benchmark dataset containing 917 questions by human workers with selected relations from Freebase.

#### Setup

Our network is written in TensorFlow [33]. We use the 300-dimensional vectors from Word2Vec [34] as our initial word embeddings. During network training, we apply RMSProp [25] as our gradient descent optimization algorithm. The RMSProp optimizer is configured with the default hyperparameters (learning rate *lr* = 1*e*^−3^, *b*_1_ = 0.9, *b*_2_ = 0.999, and *e* = 1*e*^−8^) and with the batch size set to 32. The number of iteration epochs is determined by early termination on a validation set. In the learning-to-rank procedure, we apply a random decision tree forest classifier written in scikit-learn [35].

#### Evaluation metric

Because it is the most reported and commonly used measure for the WebQuestions dataset originating from Berant [8], we use the average F1 score as our evaluation metric.

Given a list of questions [*q*_1_ … *q*_*n*_], the gold standard answer list [*g*_1_ … *g*_*n*_] and the answers [*a*_1_ … *a*_*n*_] generated by our system for each question, we compute the average F1 score across all questions as follows:

$$\begin{array}{c} \text{average}\;\text{F1}=\frac{1}{n}\sum_{i=1}^{n}F1\left(g_{i},a_{i}\right) \end{array}$$ (12)

Note that an answer (*a*_*i*_ or *g*_*i*_) is also a list of tokens, the precision, recall and the F1 over *a*_*i*_ and *g*_*i*_ are computed using the standard approach.

#### Baselines

To show the advantage of our proposed model QARDTE, we compare it with previous works by evaluating the following baseline systems:

- **Paralex** [7] selects answers by annotating similar question relations from learned relation lexicon representations.
- **Sempre** [8] is based on semantic parsing; it tries to answer questions by mapping each question to structured queries on the KB.
- **MCCNN** [5] uses a neural network model MCCNN for relation detection, and ranks answers by using a regression method based on statistical lexical features from a Wikipedia page.
- **Aqqu** [13] generates answers by finding neighbor entities over the KB and ranks the answers by calculating the text similarities between the question and the relations.

### Results and discussion

Table 1 shows the results obtained on the test set for WebQuestions with the various systems: Aqqu [13], MCCNN [5], Paralex [7] and Sempre [8]. On WebQuestions, our QARDTE system achieves improved average F1 scores compared with the baseline system Aqqu with scores of 0.549 (+1.9%) with only the improved relation detection method and 0.558 (+3.5%) with the relation detection method combined with external text resources. Furthermore, the QARDTE system obtains an average F1 score of 0.558, which outperforms the Aqqu by +3.5% and the MCCNN by +2.8%. We achieve the highest average F1 and recall scores among all of the above QA systems. Our system also achieves a precision of 0.512, a significant improvement (+6.2%) compared to Aqqu.

**Table 1:** Results on the WebQuestions test set.

Methods	Precision	Recall	Average F1
Paralex [7]	0.405	0.466	0.433
Sempre [8]	0.480	0.413	0.444
MCCNN [5]	0.537	0.550	0.543
MCCNN (w/o S-MART, Rules)	0.425	0.551	0.479
Aqqu [13]	0.482	0.611	0.539
QARDTE (w/o Wiki)	0.497	0.612	0.549
QARDTE (w/o Rel)	0.487	0.611	0.542
QARDTE	0.512	**0.613**	**0.558**

Each column presents the results obtained on the WebQuestions test set (2032 questions). The results in the table indicate that our proposed method achieves a significantly better result than the other 4 baselines. The best results achieved with our approach are marked in bold.

With different component settings, our QARDTE system performs differently: (1) When it is used with the relation detection model alone, our model shows a notable increase in precision, which indicates that the relation detection procedure helps to filter out the correct question type when performing inferences on the KB. Moreover, it works better than any other existing approach. (2) When it is used with the textual evidence extractor alone, our model achieves some increase in precision. We will present further results and explanations on this subject. (3) When it is used with both the relation detection model and external textual evidence, the performance of our model improves significantly. This indicates that combining the information obtained from relation detection and textual evidence contributes to better performance.

The MCCNN method achieves an average F1 of 0.543 by using S-MART entity linker and human-crafted question decomposition rules. However, if tested without the S-MART tools or rules, the average F1 score decreases to 0.479 (by -11.8%), and a decrease is also noted in the precision. Meanwhile, the original MCCNN method shows a 2.5% increase in precision but a notable 11.5% drop in recall compared with our method. The reason is that MCCNN differs from our system in several respects: First, MCCNN uses predefined hand-crafted question rules to decompose complex sentences. While the rules potentially improve its precision, they make it difficult to extend. Moreover, MCCNN does not handle free text such as our system; it uses only one Wikipedia page that precisely matches the topic entity of the question of interest. Such selected text resources limit the scope of identifying answers within a certain page; therefore, this approach improves precision but simultaneously hurts the method’s ability to inspect implicit relation facts and leads to notably lower recall.

Table 2 shows the results obtained on the Free917 benchmark. On the Free917 test set, our QARDTE system achieves an average F1 score of 0.663. It is superior to that of the Aqqu system at 0.627 (by +5.7%). We also achieve better precision (0.683) and higher recall (0.679) than the other baselines. However, unlike in the case of the WebQuestions results, we observe that our QARDTE+Wiki system (0.663), which tries to use external text resources via the Wikipedia extractor, shows no obvious improvement over our base system (0.659). This is because the questions in Free917 are hand-crafted and the entities in the questions are limited to those that appear in Freebase, consequently our method benefits less from using external textual evidence.

**Table 2:** Results on the Free917 test set.

Methods	Precision	Recall	Average F1
Paralex [7]	0.598	0.457	0.520
Aqqu [13]	0.646	0.647	0.627
QARDTE (w/o Wiki)	0.659	0.643	0.644
QARDTE (w/o Rel)	0.680	0.679	0.659
QARDTE	**0.683**	**0.679**	**0.663**

Each column represents the results obtained on the Free917 test set (267 questions). The best results are shown in bold.

While a modern KB contains billions of knowledge entries in the form of triples, it is impossible to predict all possible relations as the number of relations in the prediction set becomes larger. However, we note the fact that the predicates represent the hierarchical structure of the KB. Therefore, we collect a predefined subset of types as our base output relation set, and we further use the diverse category information contained in the predicate strings in our relation detection task to achieve improved performance.

Table 3 shows the relation detection performances of four different methods. Following previous work [5, 13], we use accuracy as our evaluation metric. The results presented in this table indicate that our relation detection model achieves an accuracy of 0.832 higher than those of the text matching approach, the BiCNN classification model (0.777, +7.0%) and the simple Bi-LSTM classification model (0.793, +4.8%). These findings indicate that the hierarchical category information contained in a KB often encodes domain knowledge of the entity. The information plays an important role in question type prediction for the QA task. Meanwhile, we achieve an accuracy of 0.832, and it is vastly superior to that of the MCCNN model (0.587, +41.7%). In addition, we note that the accuracy is increased (0.843, +1.3%) when using the pre-trained paraphrase layer as an input projection method. It indicates that in addition to capturing sentence grammar structures, the relation detection task may also benefit from alleviating the word lexical gap.

**Table 3:** Accuracy achieved in the relation detection sub task on the WebQuestions test set.

Model	Accuracy
Text-Matching	0.438
MCCNN	0.587
BiCNN	0.777
Bi-LSTM	0.793
QARDTE (w/o Pre)	0.832
QARDTE	**0.843**

Table 4 presents the results obtained in the relation detection task on the WebQuestions Top 5 most frequent question types in WebQuestions, comparing Aqqu and MCCNN with the model used in the QARDTE system. Our model achieves higher accuracy on each of the most frequent question types, indicating that the deep semantic representation of our model effectively learns the sentence structure information. Interestingly, it is noted that the QARDTE system with a pre-trained layer has lower accuracy than QARDTE without the pre-trained layer in some question types. We propose that it is because a layer loss exists for some structure information during projection.

**Table 4:** Accuracy achieved in the relation detection task on the Top 5 most frequent question types in WebQuestions.

Question Type	#Count	Aqqu	MCCNN	QARDTE	QARDTE(w/o Pre)
location	345	0.578	0.701	**0.974**	0.942
people	195	0.445	0.574	**0.905**	0.867
sports	129	0.446	0.585	0.862	**0.884**
government	122	0.675	0.764	0.846	**0.910**
language	101	0.818	0.627	0.926	**0.960**

In Table 5, we present some example questions from the test set. The examples indicate that our method infers a more accurate answer relation than the baseline method. For example, for the question “who is Jennifer Lawrence’s boyfriend?”, it is asking for the identity of Jennifer’s boyfriend. The baseline method provides only the answer “People”, whereas our relation detection model classifies the sentence into the “people.marriage.spouse” type, thus identifying the detailed relationship between the answer and question entities. Moreover, these example questions also show that our model captures the correct question types for questions with various expressions and different sentence structures. The first three questions ask for similar information with with different words, namely, “boyfriend”, “married to” and “wife”, whereas the next three questions ask for information with different sentence structures, namely, “language do … speak” and “people from … speak”; for both sets of questions, our model achieves equivalent and correct results.

**Table 5:** Some intuitive results of relation detection.

Simple Q1: Who is Jennifer Lawrence’s boyfriend?
Simple Q2: Who all was Richard Burton married to?
Simple Q3: Who is Martin Luther King’s wife?
Aqqu: People
MCCNN: people.marriage.spouse
QARDTE: people.marriage.spouse
Simple Q1: What language do native American Indians speak?
Simple Q2: What do people from Guam speak?
Simple Q3: What is the main language spoken in Switzerland?
Aqqu: Other
MCCNN: book.written_work (wrong result)
QARDTE: location.country.languages_spoken
Complex Q1: What kind of government did the United States have after the revolution?
MCCNN: travel.travel_destination.tourist_attractions (wrong result)
QARDTE: location.country.form_of_government
Complex Q2: What year did the Milwaukee Brewers go to the world series?
MCCNN: people.person.parents (wrong result)
QARDTE: sports.sports_team.championships

Each box presents several example questions from WebQuestions. They indicate that our method infers a more accurate answer relation than the baseline method Aqqu and MCCNN.

Meanwhile, in the second example (Simple Q1), MCCNN incorrectly classifies the question from the “language” category into “book”. We also note the results in Table 4, where MCCNN achieves the relatively lowest score in the “language” category. As most questions from the “language” category tend to have various expressions and different sentence structures, it indicates that the MCCNN model is not robust and fails to capture relation information from unseen expressions.

Additionally, we compare the performances achieved when using an LSTM network or a CNN to encode entities and predicates in our implementation. While maintaining the same neural network setup, we simply change the cells from the CNN to the LSTM type and the activation function from sigmoid to ReLU [36]. The results indicate that the LSTM network achieves better performance in this sequence classification task because of the recursive vector representations for the context of an entity, while the CNN captures only local information about each entity word. With the improved context representations in the LSTM case, we predict the question type with higher accuracy.

To show the effectiveness of the relation detection model in answering questions involving multiple relations, we seek insight regarding the results obtained in the complicated questions in the WebQuestions dataset. We use the artificial rules presented in [5] to split the WebQuestions’ test set into two categories: the former consisting of questions containing only a single relation and the latter consisting of questions containing more than one relation. Thus, the WebQuestions’ test set is split into 1,752 simple questions (86%) and 280 complex questions (14%). We then apply Aqqu, MCCNN and our proposed QARDTE method to these two test sets, and the results are presented in Table 6. As shown in the table, our method achieves improved average F1 scores compared with those of MCCNN, with scores of 0.551 (+9.8%) on the single relation questions and 0.586 (+70.3%) on the complex questions. The results indicate that our relation detection model is superior to other methods when answering complex questions with multiple relations.

**Table 6:** Average F1 results on the split WebQuestions test set.

Question Complexity	#Count	Aqqu	MCCNN	QARDTE
Single relation	1,752	0.442	0.502	**0.551**
Multiple relations	280	0.453	0.344	**0.586**

We compare the results obtained by Aqqu [13], MCCNN [5] and our QARDTE on the split WebQuestions test set (question set with a single relation and another set with multiple relations). The results reported in the table are those for the average F1 metric; we also achieve comparable results in terms of the precision and recall.

To further demonstrate the effectiveness of leveraging information from external unstructured text, we compute the average answer positions for different model setups, including the raw model without either the Bi-LSTM network or external text information and the partial model with only the external text information removed. The position of the first answer to a question that is returned by the QA system is 1, the position of the second answer is 2, etc., and the average answer position (the evaluation metric) is calculated by averaging the position number of the gold standard answers over test set questions. Table 7 presents the results. On the WebQuestions benchmark, compared with the model that does not use any external text corpus (2.781), the average answer position of the model that uses simple text statistics but does not use the deep representation Bi-LSTM model is improved to 2.357 (-15.2%), while our final model performs the best at 2.020 (-14.2%). These findings indicate that using external text data provides useful information in addition to available KB knowledge when ranking candidate answers. Our neural-network-based text inference model achieves better comprehension of the text corpus.

**Table 7:** Comparison of the average correct answer positions among different model setups.

Model	WebQuestions	Free917
QARDTE (w/o Rel, Wiki)	2.781	1.975
QARDTE (w/o Wiki)	2.357	1.966
QARDTE	**2.020**	**1.954**

**Fig 5:**
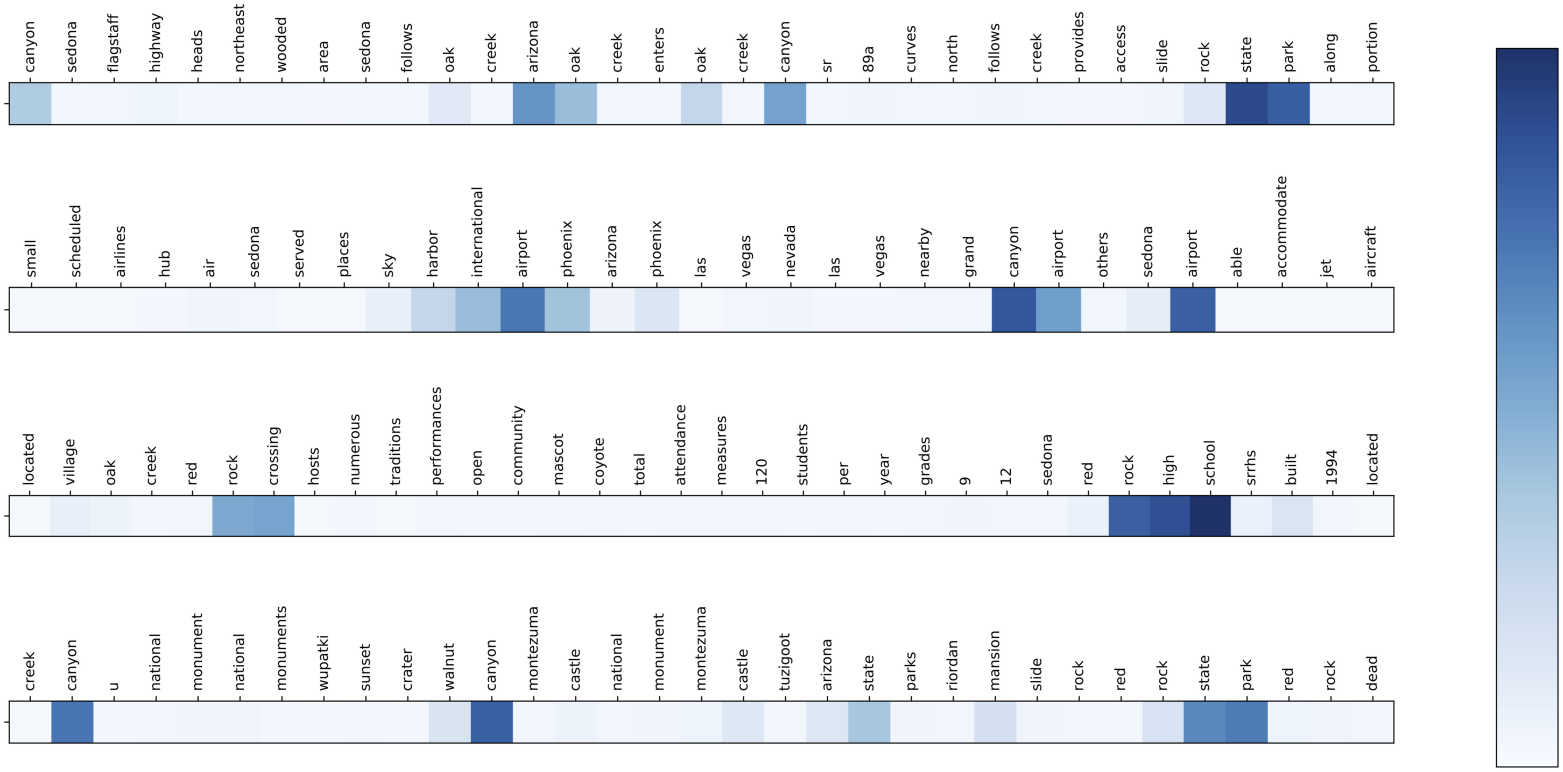
A simple example of how the attention score indicates the answers.

To show the effectiveness of our attention model, we collect the output of the attention layer when answering the question “What to see near Sedona Arizona?” from the test set. The attention score for every word in four example text snippets are demonstrated in Fig 5. Each bar represents a text snippet and each small block in a bar represents a single word. The color depth of a block represents the attention score. The deeper the color, the more attention score the word has, and the more possibility that the word is a suitable answer for the question. The standard answers to the question are “Cathedral Rock”, “Harbor Airport”, “Red Rock State Park” and “Oak Creek Canyon”. The figure shows that our model correctly assigns out higher attention scores over these answer words. Moreover, interestingly, we also note that the words “red rock high school” are not in the standard answer but are marked with a high attention score. Indeed, a “Red Rock High School” exists near Sedona in Arizona, indicating that our attention model is able to resolve implicit relations among various text pages.

Moreover, we perform Friedman’s test between the performance of our QARDTE method and the other two best baselines (Aqqu [13] and MCCNN [5]) to evaluate whether the performance variance is statistically significant.

First, on the WebQuestions dataset, we analyze the significant differences of the *F*_1_ metric. The chi-square statistic value of Friedman’s test is 169.63, larger than the Friedman critical value of 13.816 from Chi-square table (when *DF* = 2, *p* = 0.001), indicating that the variance between the different methods is significant. Furthermore, we perform a pairwise Nemenyi test between QARDTE and the baselines. The following results are obtained: QARDTE tested with Aqqu yields a p-value of 9.71e-07; QARDTE tested with MCCNN yields a p-value of 2.42e-27; and Aqqu tested with MCCNN yields a p-value of 2.32e-09. Since all of the p-values are smaller than threshold of 0.005, the variance between each pair of methods is statistically significant.

Second, the analysis on the Free917 dataset is similar. The chi-square statistic value of Friedman’s test is 97.81, indicating that the variance between the different methods is significant. Furthermore, we perform a pairwise Nemenyi test between QARDTE and the baselines. The following results are obtained: QARDTE tested with Aqqu yields a p-value of 7.85e-6; QARDTE tested with MCCNN yields a p-value of 2.96e-7; and Aqqu tested with MCCNN yields a p-value of 5.82e-09. Since all of the p-values are smaller than threshold of 0.005, the variance between each pair of methods is statistically significant.

In summary, all these results demonstrate that the performance variance between the different methods is significant. As the average accuracy of our model is higher that of Aqqu and MCCNN, our model is clearly statistically superior to those methods.

## Conclusion

In this study, we have proposed a KBQA method named QARDTE, which integrates our improved relation detection model and a textual evidence extractor to enhance the final results. The relation detection model is built on a Bi-LSTM network, which has been proven to demonstrate better performance in capturing sentence structures than any other commonly used network. We feed the network with different levels of abstraction to achieve better sentence representation. Moreover, we utilize external unstructured text to extract additional supporting evidence. Combining the information obtained from the relation detection results and the extracted textual evidence during the answer ranking process yields improved results. Experiments on two QA benchmarks, WebQuestions and Free917, show that our method achieves significant improvements compared with existing KBQA systems.

There are several possible directions for future research. First, we will look into logical inference methods to handle more complicated sentences. We can also incorporate external text for answer reasoning. Moreover, we will investigate the possibility of transfer learning. Moreover, it is possible to adapt our model to easily shift among different domain applications.
